# The effects of non-Newtonian fluid material midsole footwear on tibial shock acceleration and attenuation

**DOI:** 10.3389/fbioe.2023.1276864

**Published:** 2023-12-13

**Authors:** Enze Shao, Qichang Mei, Julien S. Baker, István Bíró, Wei Liu, Yaodong Gu

**Affiliations:** ^1^ Faculty of Sport Science, Ningbo University, Ningbo, China; ^2^ Auckland Bioengineering Institute, The University of Auckland, Auckland, New Zealand; ^3^ Centre for Population Health and Medical Informatics, Hong Kong Baptist University, Kowloon, Hong Kong SAR, China; ^4^ Faculty of Engineering, University of Szeged, Szeged, Hungary; ^5^ Department of Radiology, Ningbo No. 2 Hospital, Ningbo, China

**Keywords:** non-Newtonian flow, ethylene vinyl acetate, shock acceleration, biomechanics, tibial

## Abstract

**Introduction:** Given the possibility of higher ground temperatures in the future, the pursuit of a cushioning material that can effectively reduce sports injuries during exercise, particularly one that retains its properties at elevated temperatures, has emerged as a serious concern.

**Methods:** A total of 18 man recreational runners were recruited from Ningbo University and local clubs for participation in this study. Frequency analysis was employed to investigate whether there is a distinction between non-Newtonian (NN) shoes and ethylene vinyl acetate (EVA) shoes.

**Results:** The outcomes indicated that the utilization of NN shoes furnished participants with superior cushioning when engaging in a 90° cutting maneuver subsequent to an outdoor exercise, as opposed to the EVA material. Specifically, participants wearing NN shoes exhibited significantly lower peak resultant acceleration (*p* = 0.022) and power spectral density (*p* = 0.010) values at the distal tibia compared to those wearing EVA shoes. Moreover, shock attenuation was significantly greater in subjects wearing NN shoes (*p* = 0.023) in comparison to EVA shoes. Performing 90° cutting maneuver in NN shoes resulted in significantly lower peak ground reaction force (*p* = 0.010), vertical average loading rate (*p* < 0.010), and vertical instantaneous loading rate (*p* = 0.030) values compared to performing the same maneuvers in EVA shoes.

**Conclusion:** The study found that the PRA and PSD of the distal tibia in NN footwear were significantly lower compared to EVA footwear. Additionally, participants exhibited more positive SA while using NN footwear compared to EVA. Furthermore, during the 90° CM, participants wearing NN shoes showed lower PGRF, VAIL, and VILR compared to those in EVA shoes. All these promising results support the capability of NN footwear to offer additional reductions in potential injury risk to runners, especially in high-temperature conditions.

## 1 Introduction

The increased public participation in sports over the last few decades has led to a rise in sport-related pathologies among both recreational and competitive athletes (Knowles et al., 2006; Marconcin et al., 2023). In most sports, athletes commonly employ a heel-toe gait pattern when landing during sports performance (Anderson et al., 2020). In such instances, pronation at the subtalar joint (STJ) occurs from the moment of heel strike to midstance (Leung et al., 1998; Phan et al., 2018). According to Klingman et al. (1997), the pronation of the STJ is correlated with knee flexion and internal tibial rotation. This sequence of movements assumes a pivotal role in dampening the shock when the heel comes into contact with the ground. It is conjectured that the compensatory internal rotation of the femur might aid in preserving alignment during knee extension (Tiberio, 1987; Boutris et al., 2018). Nevertheless, over the long term, athletes and sports performers face an elevated likelihood of encountering discomfort, or worse, injuries to the patellofemoral joint, which has the potential to undermine athletic prowess (Deng et al., 2022). Furthermore, it has been reported that the majority of chronic injuries occurring in the lower limbs are intricately linked to cumulative loading (Van Gent et al., 2007). Particularly pertinent in the realm of athletics, it is noteworthy that between 35% and 49% of fatigue fractures manifest in the tibia (Crossley et al., 1999; Bennell et al., 2004). Numerous variables may indeed exert an impact on bone remodeling, thereby influencing the performance of fatigued bones. What is evident is that biomechanics elucidates the extent of mechanical loading endured by the bone throughout the course of a movement (Levenston and Carter, 1998; Logerstedt et al., 2022). Upon impact with the ground, the velocity of the foot decelerates to zero, leading to the generation of great ground reaction forces (GRF) (Whittle, 1999). This alteration in momentum leads to the compressive loading of lower extremities, resulting in an impact shock delivered via the musculoskeletal system. Consequently, local segment peak accelerations occur at progressively delayed intervals (Derrick, 2004; Reenalda et al., 2019). The correlation between tibial acceleration (TA) and bone strain remains enigmatic and may be intricate due to the influence of localized muscle forces.

It is noteworthy that measuring peak TA using a device directly attached to the tibia proves to be an effective method for revealing plausible correlations with essential GRF parameters (Hennig and Lafortune, 1991). Moreover, due to their convenience, an increasing number of studies are employing wearable inertial measurement units (IMUs) to collect TA data. This approach has provided valuable insights into the mechanisms contributing to the understanding of stress fractures and joint motion injuries (Yong et al., 2018; Milner et al., 2022), utilizing technological approaches such as frequency analysis (Xiang et al., 2022) and machine learning (Tenforde et al., 2020).

When a runner’s heel strikes the ground, the rapid deceleration creates a shock wave that travels from the foot to the torso and through the entire skeletal system. The energy of this shockwave is assimilated by various components, encompassing footwear, running surfaces, muscles, bones, and other structural tissues (Derrick et al., 1998). This process of absorbing impact energy, consequently diminishing the amplitude of the shock wave between the foot and the head, is denoted as shock attenuation (SA). In addition to internal forces (Richards et al., 2018), SA and the magnitude of impact acceleration emerge as two pivotal variables scrutinized in running research (Milner et al., 2006), owing to their conjectured correlation with prospective injuries. Researchers believe that in order to minimize damage to proximal structures, SA can be achieved through an interplay of passive and active mechanisms (Derrick et al., 1998; Milner et al., 2006; Zadpoor and Nikooyan, 2012). Based on the above hypotheses, previous studies have explored several factors, such as the performance of eccentric muscle contractions (Mizrahi et al., 2000), running speed (Sheerin et al., 2019), exercise fatigue interventions (Flynn et al., 2004), running surface (Boey et al., 2017), and running shoes (Xiang et al., 2022), while observing and comparing changes in TA. To be definitive, subjects experienced highly significant changes in TA at different running speeds, during different motion interfaces, and under enhanced eccentric muscle contractions. Nevertheless, there is some controversy surrounding the effect of different footwear types on TA (Cheung et al., 2006; Sinclair et al., 2013). These disputes mostly arise due to the variations in footwear conditions and the distinct production process requirements across different footwear companies. Indeed, to ascertain the effect of footwear on tibial impact, more definitive information is required, considering factors such as the exercise environment, movement standards, and other relevant variables. It is worth noting that during exercise, the temperature of the running shoe will naturally increase, which could potentially lead to a deterioration in its cushioning properties (Kinoshita and Bates, 1996).

Indeed, this is often a factor that is overlooked by researchers. Amidst the ongoing escalation of average global temperatures, particularly in the realm of extreme climatic conditions, running footwear may pose an augmented risk of injury to the runner as it experiences heightened temperatures during outdoor exercise engagement (Ebi et al., 2021). This emphasizes the importance of investigating and addressing the impact of temperature changes on running shoe properties for the safety and well-being of athletes. Given the potential for higher ground temperatures in the future, the quest for a cushioning material that can effectively reduce sports injuries during exercise, particularly one that retains its properties at elevated temperatures, has emerged as an urgent necessity. Drawing from the findings of impact dynamics and materials development studies (de Goede et al., 2019), it has been demonstrated that non-Newtonian (NN) fluid materials possess the capability to effectively manage impact force or acceleration decay scenarios. Therefore, in the development of sports protective equipment (Hrysomallis, 2009; Schmitt et al., 2010; La Fauci et al., 2023), designers utilize the viscoelastic and permanent deformation properties of NN fluid to minimize the impact damage of solids on the human body. Undoubtedly, the specific temperature may exert a discernible influence on the functionality of non-Newtonian fluid materials. The effectiveness of these materials in SA and protective equipment largely depends on maintaining the appropriate temperature during their usage. Past investigations have demonstrated that a substantial elevation in the temperature of EVA footwear can lead to a notable surge in the vulnerability to lower limb injuries when individuals partake in physical activities (Shariatmadari et al., 2010). It's worth noting that M. Hojjat et al. observed that the rheological properties of non-Newtonian fluids exhibited shear-thinning behavior following an increase in temperature (Hojjat et al., 2011). Therefore, the incorporation of materials with NN fluid properties into footwear has the potential to provide protection for athletes during outdoor running or sports activities, particularly when the temperature of the footwear rises. By leveraging the unique characteristics of NN fluid, footwear can better adapt to varying impact conditions, ensuring enhanced SA, and reducing the risk of injuries for athletes.

## 2 Materials and methods

In this section, we primarily delineate the research hypothesis of this endeavor, elucidate the precise steps undertaken for data acquisition, and expound upon the methodologies employed for data processing throughout the experiment. Subsequently, the statistical analysis approach is detailed. In essence, two IMUs were affixed to the anterolateral distal aspects of the tibia in the subjects. This was carried out to juxtapose the TA and mechanical attributes of the subjects when executing a 90°CM while clad in both EVA and NN footwear.

### 2.1 Working research hypothesis

The research hypothesis of this study was that there would be significant differences in tibial acceleration and attenuation when participants wore NN shoes during outdoor running sessions for extended periods, as compared to EVA shoes. By examining the impact attenuation and SA of these shoes, this study aims to assess their effectiveness in providing protection and comfort to athletes during outdoor activities in elevated temperatures.

### 2.2 Participants

Considering the potential differences in TA and impact attenuation due to gender (Sinclair et al., 2012), a total of 18 man recreational runners (age: 24.32 ± 1.20 years, height: 1.78 ± 0.04 m, mass: 64.61 ± 1.22 kg, BMI: 20.22 ± 0.41 kg/m^2^) were recruited from Ningbo University and local clubs for participation in this study. A statistical power analysis was performed employing G*Power software, employing a moderate effect size to mitigate the risk of a type II error and ascertain the minimum number of participants requisite for this inquiry (Erdfelder et al., 1996). The input parameters for this experiment were tailored as follows: the effect size was set at 0.4, the significance level (Alpha) at 0.05, the test efficacy (Power) at 0.8, the number of measurements at 3, and the Nonsphericity at 0.5. The sample size employed in this study was adequate to yield statistical power exceeding 80%. The inclusion criteria for participants consisted of recreational level runners, right leg-dominant, and habitual rearfoot strikers. Recreational runners are defined as individuals who engage in running activities 2–4 times per week and cover a distance of at least 20 kilometers per week (Liu et al., 2020). To be eligible for the experiment, the recruited runners had to exhibit no lower limbs injuries or foot deformities in the 6 months preceding the testing Before data collection, all subjects were fully familiarized with testing procedures and different running shoes. All data collection was obtained at the same time of day to minimize the effects of diurnal variation on experimental results. Additionally, all participants were provided with the option to withdraw from the experiment at any stage of testing, and written informed consent was obtained from each participant before commencement of the study. The Ethics Committee of the Ningbo University Research Institute granted approval for this study (RAGH202208193312), which was conducted in adherence to the principles of the Declaration of Helsinki.

### 2.3 Experiment protocol

The test was divided into three parts, with the first part aimed at determining whether all subjects met the inclusion criteria. In accordance with a previous study, the dominant foot was determined to be the right foot based on the single-legged hop for distance test. The rearfoot strike pattern was characterized by employing the strike index, denoting the center of pressure within the initial 0%–33% of the foot length at contact, as measured by the Footscan^®^ pressure plate (Rsscan International, Olen, Belgium).

The second part of the test consisted of an outdoor five-kilometer even-paced running session in EVA and NN shoes. Participants engaged in a standardized warm-up routine, comprising a 5-min jog at a self-selected pace on a motorized treadmill, along with several stretching exercises. The participants were blinded to the type of shoe used during testing, and shoes were assigned to participants in a random sequence. Afterwards, the participants wore EVA or NN footwear for a 5-kilometer outdoor run, with 18 participants trained in an average outdoor temperature of 38.12 ± 1.20 degrees Celsius. The run was completed at an average pace of 10.8 ± 0.5 km/h. Following 3 days of rest, the subjects once again engaged in a five-kilometer outdoor run, this time wearing EVA or NN shoes, under similar temperature conditions.

The third part of the test involved subjects wearing EVA and NN shoes and collecting the surface temperature of the running shoes immediately after completing a 5-kilometer outdoor run. Immediately after the outdoor run, tibial impact testing was conducted in the laboratory. This involved the simultaneous collection of the subject’s vertical ground reaction force and accelerometer data. Indeed, the frequent requirement to perform unexpected or anticipated cutting maneuver (CM) while running or engaging in outdoor activities poses a significant risk of injury (Nagano et al., 2016). Previous studies have confirmed that there is a greater risk during 90° CM. Therefore, in this study, 90° CM was chosen as the impact test action. This was used to assess the effects of footwear and other variables on tibial impact during lateral movements, which were common in various sports and outdoor activities.

**FIGURE 1:**
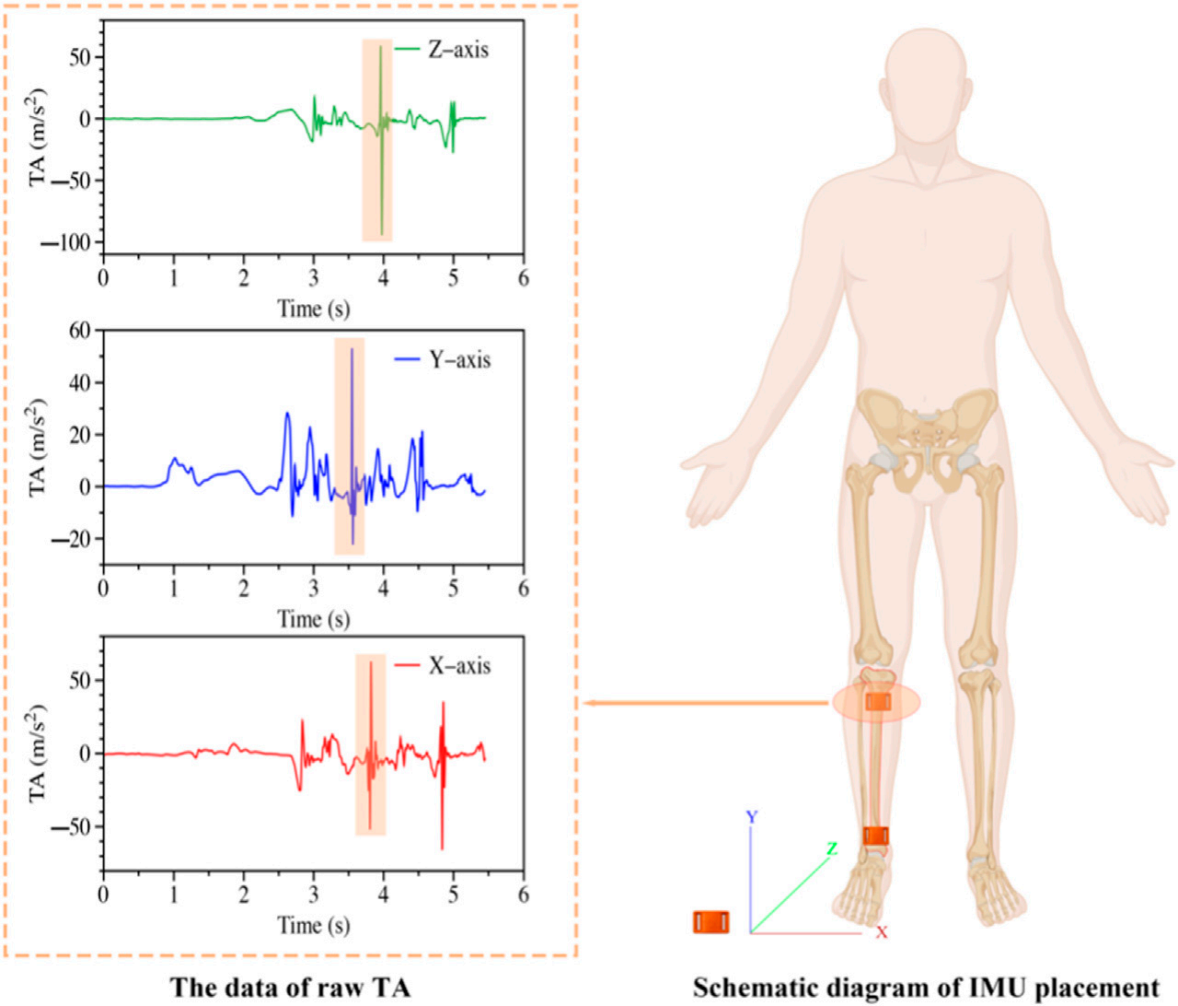
Data of raw TA (left) and the IMU fixed position (right). The orange shaded portion of the TA raw data represents the acceleration data recorded during the CMs.

The IMUs (IMeasureU V1, Auckland, New Zealand; dimensions: 40 mm × 28 mm × 15 mm, weight: 12 g, resolution: 16 bit) were affixed to the proximal and distal anteromedial regions of the tibia on the dominant leg of each participant using straps. Precisely, two Inertial Measurement Units (IMUs) were situated on the anterior medial aspect of the tibia, exactly 2 centimeters proximal to the ankle and 3 centimeters from the tibia’s proximal end (Laughton et al., 2003), and securely fastened with athletic tape up to an acceptable tension level. The vertical axis of the accelerometer was aligned with the tibia (as depicted in Figure 1) (Tenforde et al., 2020). The tension of the belt was carefully adjusted to a level where the acceleration traces for a given impact force remained insensitive to the accelerometer attachment force. This measure was taken to ensure the reproducibility of the data collected during the study (Mizrahi and Susak, 1982). Subsequently, participants performed 90° CM at the corner of the laboratory’s six-meter track, maintaining a running speed consistent with outdoor conditions. IMUs were synchronized with an embedded AMTI force platform (AMTI, Watertown, MA, United States), which was placed in the center of the pathway.

A single-beam electronic timing gate (Brower Timing Systems, Draper, UT, United States) was employed to record and control the subject’s running speed. During the experiment, each participant performed 90° CM 10 times, and after completing each set of CM, the subject was given a 1-min rest period before the next set. This approach ensures sufficient recovery time between trials to minimize fatigue and maintain the consistency of data collection.

### 2.4 Footwear characteristics

**FIGURE 2:**
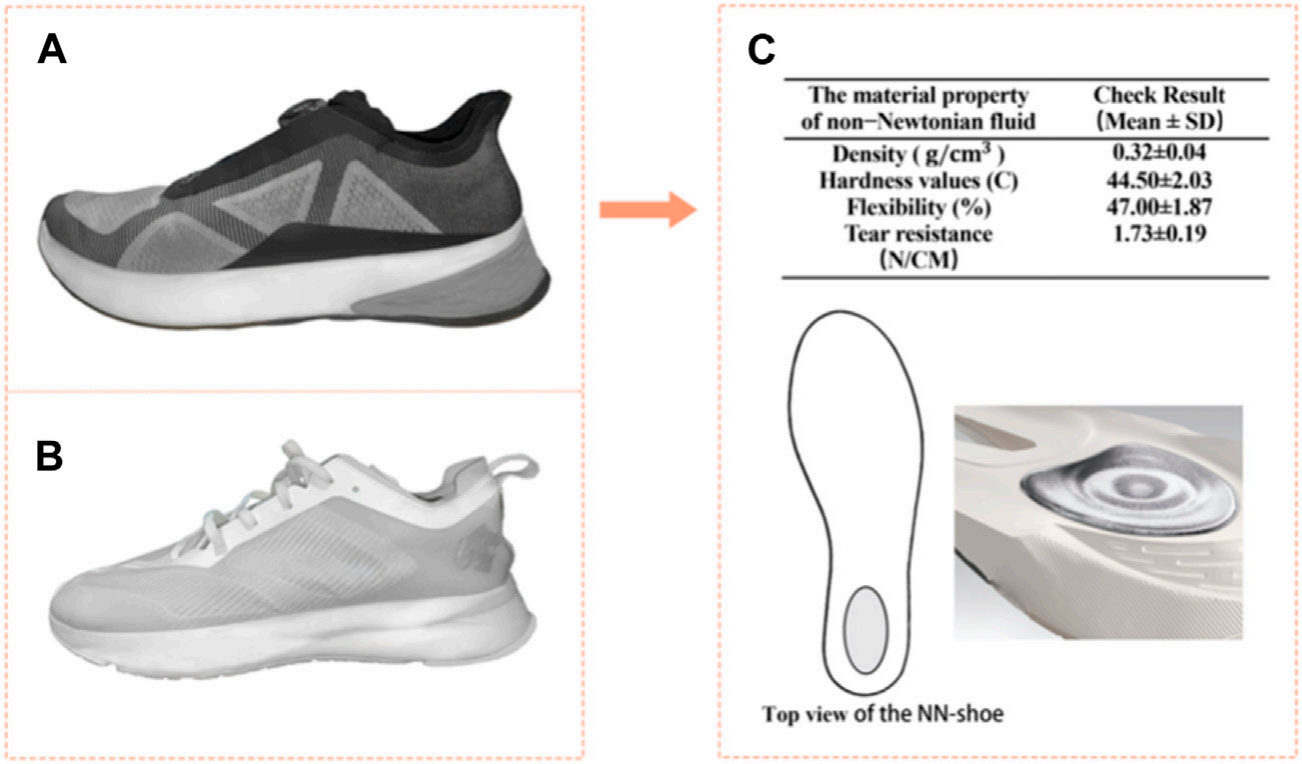
Experimental shoes were used by the participants. **(A)** indicate the non-Newtonian fluid (NN) shoe; **(B)** indicate the EVA shoe; **(C)** indicate the material property of non-Newtonian fluid as well as the location of cushion materials within the midsole.

NN shoes (as shown in Figure 2A) were developed and manufactured by the Japanese manufacturer (Descente Ltd., Kabushiki−gaisha Desanto, Osaka, Japan). The same manufacturer, Descente Ltd., also produced the EVA shoes utilized in this investigate (as shown in Figure 2B). In the NN shoes, the NN material was placed in a triangular area on the heel of the midsole (as shown in Figure 2C). The NN materials and preparation methods used in the footwear have been disclosed in a patent of invention (patent publication No: CN116285389A), and the material properties comply with the national requirements for the production of the footwear in question (Figure 2C).

### 2.5 Data collection and processing

AMTI was utilized to collect GRF data during the CM at a frequency of 1,000 Hz. As for the GRF of CM, a vertical threshold of 20N was utilized to detect foot strike and toe-off events, thus delineating the stance phase (Yu et al., 2021). To reduce the influence of random noise, the GRF data were filtered using a low-pass, second-order Butterworth filter with a cut-off frequency set at 20 Hz (David et al., 2018). All of the GRF magnitudes were scaled to body mass. This study addresses the vertical GRF associated with the 90° CM performed by the subjects. The key parameters to be compared and analyzed are the peak ground reaction force (PGRF), vertical average loading rate (VALR), and vertical instantaneous loading rate (VILR) (Jiang et al., 2021). These parameters are essential in understanding the impact forces experienced during the CM and their potential implications for injury risk and performance.

The IMUs collected TA data at a frequency of 500 Hz while the subjects were wearing both types of footwear.

To eliminate a linear trend, the raw data signal underwent a process of subtraction, wherein a least-squares best-fit line was deducted from it (Shorten and Winslow, 1992). Subsequently, the collected data was filtered using a second-order Butterworth low-pass filter with a cutoff frequency set at 60 Hz (Hennig and Lafortune, 1991). While axial acceleration is commonly reported, recent recommendations suggest evaluating resultant acceleration (RA) (Sheerin et al., 2019; Milner et al., 2020). Calculated RA was completed using the following formula:

$$\text{RA }=\sqrt{x^{2}+y^{2}+z^{2}}$$

x, y, and z represent the acceleration variations in the coronal, sagittal, and transverse planes of the IMU, respectively (shown in Figure 1, right).

Peak resultant acceleration (PRA) was identified as the peak occurring between 50% and 60% of stance. Time-domain and frequency parameters from the accelerometers at both ends of the tibia were computed using a custom MATLAB R2018b program (The MathWorks, Natick, MA, United States). Time-domain parameters were determined based on the last stance phase performed by each participant. To achieve this, the power spectrum was analyzed by converting the time-domain signal to frequency using a discrete fast Fourier transform (FFT). The unfiltered TA data from each stance stage underwent detrending and were subsequently extended with zeros to achieve a total of 2048 data points, ensuring periodicity. To determine the power of the standing-phase TA in the frequency domain, the power spectral density (PSD) was calculated using a square window (Shorten and Winslow, 1992). The PSD analysis was conducted within the frequency range from 0 to the Nyquist frequency (FN) and then normalized into 1 Hz bins (Gillespie and Dickey, 2003). A transfer function has been previously used to determine the degree of SA in human running by calculating the ratio of each frequency bin the distal and proximal tibia signal (i.e., the transmissibility of each frequency component) (Hamill et al., 1995). The transfer function was computed across all frequencies ranging from 0 to FN, aiming to ascertain the extent of SA taking place between the distal and proximal tibia. This calculation was achieved by:

$$\text{Shock }\text{attenuation }=10\times \log_{10}\left({PSD}_{p\_tibia}/{PSD}_{d\_tibia}\right)$$

At each of the frequencies, the transfer function determined the gain or attenuation, measured in decibels, between the distal and proximal tibia signals. Positive values signified a gain, indicating an increase in signal strength, while negative values denoted attenuation, representing a reduction in signal strength.

### 2.6 Statistical analysis

All discrete feature data, including PGRF, VALR, VILR, and PRA, are presented as mean ± standard deviation. A one-way repeated measures analysis of variance (ANOVA) was conducted to assess the impact of shoe condition (differentiating between NN and EVA footwear) on the discrete data. A significance level of *p* < 0.05 was considered acceptable. The *post hoc* pairwise comparison was conducted using the Bonferroni correction, which adjusted the significance level to *p* < 0.033. The Shapiro-Wilk test was utilized to evaluate the normal distribution of RA, PSD, and SA during the 90° CM. Following the results of the normality test (Pataky et al., 2015), SPM1D or SNPM1D analysis was conducted to examine the differences in RA, PSD, and SA when wearing different footwear, respectively. For this analysis, MATLAB R2018b (The MathWorks, Natick, MA, United States) was used to perform all the statistical calculations.

## 3 Results

Following the completion of the 5-kilometer run, the midsole temperature of the NN footwear escalated from 22.53°C ± 0.43°C to 54.84°C, while the midsole temperature of the EVA footwear rose from 22.46°C ± 0.52°C to 50.87°C. As indicated in Table 1, there was no statistically significant difference in the PRA of the proximal tibia between the two types of footwear (*p* = 0.270). Additionally, the time of foot contact with the ground during the 90° CM was nearly similar for both footwear conditions (*p* = 0.550). The ANOVA revealed a statistical difference in the PRA of the distal tibia between the two shoe conditions (*p* = 0.022). In comparison to EVA shoes, subjects exhibited significantly lower values for PGRF (*p* = 0.020), VALR (*p* < 0.010), and VILR (*p* = 0.030) when performing 90° CM while wearing the NN footwear.

**TABLE 1:** The ANOVA results in the discrete characteristics of the two types of shoes {data were presented in mean [standard deviation (SD)]}.

Discrete characteristics	NN	EVA	F−Value	*p*−Value
PRA at distal tibia (g)	14.21 (1.17)	15.37 (2.28)	1.423	**p = 0.022**
PRA at proximal tibia (g)	7.99 (3.61)	10.45 (1.94)	2.367	*p* = 0.270
PGRF (N/kg)	2.43 (0.19)	2.61 (0.30)	5.662	**p = 0.010**
VALR (N/kg/s)	85.23 (23.14)	95.16 (28.02)	12.511	**p < 0.010**
VILR (N/kg/s)	154.27 (28.33)	160.24 (37.22)	17.312	**p = 0.030**
Contact Time (s)	0.24 (0.03)	0.23 (0.02)	0.932	*p* = 0.550

The bold values are meant to indicate that there is statistical significance in the group.

**FIGURE 3:**
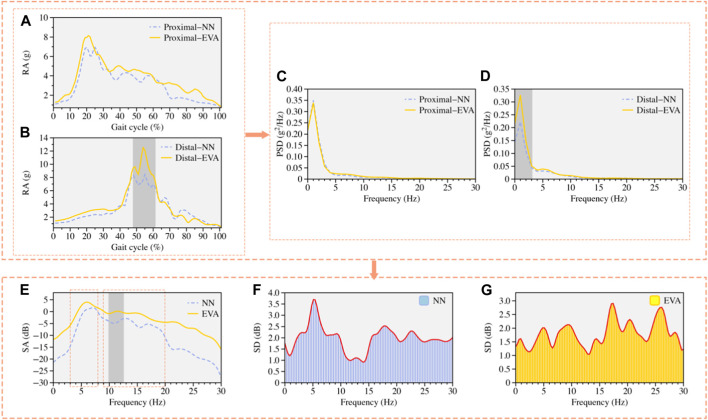
The results of the TA processing in the time and frequency domains for the two types of footwear (NN and EVA) were plotted. These encompass the following variables: RA of the proximal tibia **(A)**, RA of the distal tibia **(B)**; PSD of the proximal **(C)** and distal **(D)** tibia; SA from the distal tibia to the proximal tibia **(E)**, and changes in SD of SA in the tibia for non-Newtonian **(F)** and EVA **(G)** footwear. **(A) (B) (C) (D) (E)** The curves depicted in the figures represent the mean changes for the corresponding variables. The deeply shaded areas correspond to the station phases or frequency bands wherein a significant main effect was detected based on the SNPM1D test.

Based on the results of the Shapiro-Wilk test, it was determined that RA, PSD, and SA did not follow a normal distribution (*p* < 0.05). As a result, these non-normally distributed data were analyzed using the SNPM1D method. As depicted in Figure 3, when comparing the changes in RA with different footwear (Figures 3A, B), it was observed that RA in the distal tibia was significantly higher in the peak region when wearing EVA footwear compared to NN footwear (*p* = 0.011, 47%–61 stage). As depicted in Figures 3B, C, the PSD in the lower frequency range of the distal tibia exhibited a statistically significant difference between the NN and EVA footwear (*p* = 0.010). The EVA footwear exhibited significantly greater PSD power at low frequencies compared to the NN footwear. As shown in Figures E, F, the NN footwear demonstrated significantly greater SA at higher frequencies (10–13 Hz) compared to the EVA footwear (*p* = 0.023). However, there was no significant variation in SA at lower frequencies (3–8 Hz) between the two types of footwear (as shown in Figures E, G).

## 4 Discussion

The primary objective of this study was to examine the alterations in time and frequency domain attributes of TA in subjects wearing different types of footwear after completing a 5-kilometer run in higher temperature conditions. The research focused on comparing the impact and SA performance of running shoes embedded with NN footwear and EVA running shoes. Through the analysis of TA in both time and frequency domains, the study aimed to elucidate the potential advantages of using NN footwear in offering functional cushioning and decreasing sports-related injuries during exercise in hot environments.

These findings indicate that both types of footwear experienced significant temperature increases during the running activity in higher temperature conditions, with the NN footwear showing a slightly higher temperature rise compared to the EVA footwear. Based on the 90° CM test conducted after the temperature change of the footwear, the study found that the PRA at the distal tibia was significantly lower in NN shoes compared to EVA shoes. Additionally, the PGRF, VALR, and VILR were also significantly lower in NN shoes than in EVA shoes, as indicated in Table 1.

An ideal approach for precisely assessing injury risk in runners would involve direct in-vivo measurement of bone strain. Nevertheless, this approach is invasive and impractical for routine use, as it necessitates surgical implantation of strain gauges or other invasive methods (Liu et al., 2009). Consequently, it has become a widely employed approach to attach accelerometers to the body segments of interest to calculate the corresponding impact forces (Gruber et al., 2014). Previous studies have aimed to observe the protective impact of running shoes on the human body during exercise. This has been achieved by investigating the effect of various footwear conditions, such as traditional footwear (Sinclair and Sant, 2017), minimalist footwear (Sinclair et al., 2013), and customized footwear (Laughton et al., 2003) on alterations in TA. This study builds on previous research and contributes to the understanding of how global temperature changes may impact the performance and function of footwear.

It was readily evident from the time and frequency domain results for TA that distinct footwear conditions led to alterations in distal TA. In the time-domain analysis, the waveforms of TA exhibited differences in their shape and amplitude, indicating variations in impact forces and loading patterns on the tibia between the two types of shoes. These changes are crucial indicators of how the footwear’s cushioning and SA properties influenced the tibial response to impact forces during the CM. Previous studies have established that RA effectively mitigated the impact of accelerometer misalignment and accounted for loading forces across all three axes (Norris et al., 2014). The joint kinematics observed at impact, including greater heel vertical velocity, increased lower leg angle, reduced knee flexion angle, and an extended stride length, contribute to higher TA levels upon impact (Potthast et al., 2010). However, throughout the experimental process, we maintained consistency requirements for all factors except the footwear condition. Thus, it is highly plausible that the decreased PRA of the distal tibia in heel strike mode could suggest that the NN footwear might have offered superior mechanical cushioning during braking, in conjunction with the active mechanism of the body’s muscular contraction. Compared to NN shoes, EVA shoes resulted in greater TA power magnitude in the lower range. This aligns with previous studies indicating that employing heel strikes leads to increased power amplitude at lower frequencies (Oakley and Pratt, 1988). As the heel strike mode of running involves reduced knee flexion and velocity, it induces an elevation in the power amplitude of the tibial signal below 10 Hz. In other words, engaging in running with NN shoes may lead to an increase in a specific knee flexion angle, where a compliant knee assumes a more significant role in active shock attenuation during rearfoot running compared to the ankle. No significant alteration in impact force and impact attenuation was observed in the proximal tibia when wearing different footwear, which could be attributed to the mechanical cushioning function of the footwear predominantly acting from the ankle to the distal tibia (Schütte et al., 2018). The mechanical cushioning function of footwear, typically concentrated in the midsole area, proves particularly effective in mitigating impact forces transmitted to the distal tibia. When the foot contacts the ground during running, the cushioning materials in the midsole efficiently absorb and disperse impact energy, thereby reducing the forces exerted on the distal tibia and safeguarding the lower limb against excessive loading.

Consequently, for knee injury prevention, it becomes crucial to consider additional measures that focus on mechanical cushioning or training tools that enhance active cushioning. This is especially important as the knee plays a more substantial role in shock absorption of external forces compared to the ankle (Hamill et al., 2014). Furthermore, NN footwear exhibited a greater SA effect in the higher frequency domain when compared to EVA footwear. This indicates that the tibia is able to dissipate a higher amount of shock load with NN footwear (Gruber et al., 2014). SA can be considered an accelerometry variable that may offer a more precise reflection of impact severity, particularly when the effective mass is not constant. The extent of required attenuation can modify the runner’s kinematics and performance, making it a critical factor to consider (Derrick, 2004). Prior research has validated that higher PGRF, VAIL, and VILR may elevate the risk of injury in runners (Logan et al., 2010). Conversely, higher impact and loading rates could suggest that the footwear provides insufficient cushioning, thereby increasing the risk of lower extremity injuries. The findings presented in Table 1 demonstrated that, with no alteration to other footwear properties, the footwear with only the addition of NN material exhibited substantial changes in PGRF, VAIL, and VILR. Indeed, the significant changes in PGRF, VAIL, and VILR illustrated the positive impact of NN material on the footwear’s shock-absorbing capabilities. The study emphasizes the potential of employing advanced materials in sports footwear design to enhance athlete performance and reduce the risk of injury, particularly in situations where elevated temperatures may affect the cushioning properties of the footwear.

In the process of interpreting the results, it is crucial to acknowledge several limitations inherent in the current study. Specifically, only male runners who habitually employ a rearfoot strike pattern were recruited as participants. Hence, it is important to note that these findings are not applicable to habitual mid- and forefoot strike runners. Ultimately, it's imperative to note that this study was conducted within a controlled laboratory setting and the impact of similar training in runners’ more natural environment remains unknown. In addition, future studies should also consider the biomechanical effects associated with gender differences.

## 5 Conclusion

The study found that the PRA and PSD of the distal tibia in NN footwear were significantly lower compared to EVA footwear. Additionally, participants exhibited more positive SA while using NN footwear compared to EVA. Furthermore, during the 90° CM, participants wearing NN shoes showed lower PGRF, VAIL, and VILR compared to those in EVA shoes. All these promising results support the capability of NN footwear to offer additional reductions in potential injury risk to runners, especially in high-temperature conditions.

## Data Availability

The original contributions presented in the study are included in the article/Supplementary Material, further inquiries can be directed to the corresponding authors.
